# Hydromethanolic Extracts from *Adansonia digitata* L. Edible Parts Positively Modulate Pathophysiological Mechanisms Related to the Metabolic Syndrome

**DOI:** 10.3390/molecules25122858

**Published:** 2020-06-21

**Authors:** Stefania Cicolari, Marco Dacrema, Arold Jorel Tsetegho Sokeng, Jianbo Xiao, Achille Parfait Atchan Nwakiban, Carmen Di Giovanni, Cristina Santarcangelo, Paolo Magni, Maria Daglia

**Affiliations:** 1 Department of Pharmacological and Biomolecular Sciences, Università degli Studi di Milano, 20133 Milan, Italy; stefania.cicolari@unimi.it; 2 Department of Pharmacy, University of Naples Federico II, Via Domenico Montesano 49, 80131 Naples, Italy; marco.dacrema@unina.it (M.D.); carmen.digiovanni@unina.it (C.D.G.); cristina.santarcangelo@unina.it (C.S.); 3 Department of Drug Sciences, Medicinal Chemistry and Pharmaceutical Technology Section, University of Pavia, Viale Taramelli 12, 27100 Pavia, Italy; aroldjorel.tseteghosokeng01@universitadipavia.it; 4 International Research Center for Food Nutrition and Safety, Jiangsu University, Zhenjiang 212013, China; jianboxiao@umac.mo; 5 Department of Biochemistry, Faculty of Science, University of Dschang, PO Box 67, Dchang, Cameroon; achilestyle@yahoo.fr; 6 IRCCS MultiMedica, 20099 Sesto San Giovanni, 0039 02 Milan, Italy

**Keywords:** *Adansonia digitata* L., baobab edible parts, digestive enzyme inhibition, ACE inhibition, HMG-CoA reductase inhibition, SW-872 preadipocytes

## Abstract

Metabolic syndrome includes a cluster of risk factors for many pathological conditions, including hyperglycemia, abdominal obesity, hyperlipidemia, and hypertension. *Adansonia digitata* L. (also known as baobab) is used in traditional African Medicine and recent studies showed that it improves the metabolism of carbohydrates and lipids. The aim of this study is to investigate the mechanisms of action associated with the beneficial effects of extracts from the edible parts of baobab (fruit pulp, leaves, raw and toasted seeds), evaluating their inhibitory activity against: alpha-amylase, alpha-glucosidase, angiotensin-converting enzyme, 3-hydroxy-3-methyl-glutaryl-coenzyme A reductase, and pancreatic lipase. Baobab fruit pulp and leaf extracts resulted to be the most active ones and were then tested on the differentiation process of SW-872 human liposarcoma cells to mature adipocytes. The addition of these latter extracts did not affect triglyceride accumulation, indicating a neutral impact on this parameter. The findings here reported help to explain the growing amount of evidence on the biological properties of baobab and provide suggestions about their use in food and nutraceutical fields.

## 1. Introduction

Metabolic syndrome (MS) is a condition of low-grade chronic inflammation generated by a complex interplay between genetic and environmental factors. Generally, it provokes a cluster of risk factors that include hyperglycemia, abdominal obesity, hyperlipidemia, and hypertension. It may evolve in severe systemic effects such as stroke, coronary heart disease, myocardial infarction, type 2 diabetes mellitus, cancer, and other diseases, which may lead to death [1]. The prevalence of MS in the different regions of the world ranges from under 10% to as much as 84% [2,3,4]. Aetiology of MS is mainly linked with unhealthy lifestyle; thus, its incidence is relatively common. MS prevention might be achieved by adopting lifestyle changes (weight loss and increased physical activity) and following a healthy diet characterized by high consumption of fruits, vegetables, and whole grains and a low intake of *trans* fatty acids, cholesterol, and salt. Considering that maintaining a healthy lifestyle is an everyday challenge and in some cases a simple change in lifestyle may be not effective, specific pharmacological treatments are often prescribed (i.e., statins to decrease hypercholesterolemia, renin-angiotensin-aldosterone system inhibitors for hypertension, metformin or sodium/glucose cotransporter 2 inhibitors or glucagon-like peptide 1 receptor agonists (GLP-1RAs) for hyperglycemia, and the GLP-1RA liraglutide to reduce obesity) [5]. Nevertheless, these drugs can exert serious side effects that can lead to low therapy compliance, enhancing the risk to develop severe forms of the above-mentioned pathologies. Moreover, a large portion of possible patients is not fully eligible for drug treatment and may benefit from different approaches. In this context, the interest in functional foods and food supplements able to reduce modifiable MS risk factors, such as anti-hypercholesterolemic and anti-hyperglycemic agents, continues to increase [6].

*Adansonia digitata* L., known as baobab, is a plant indigenous to Africa. Its edible parts have a significant and consolidated history of human consumption in this geographical area, where baobab is used both as a food and in traditional African medicine against some disorders, including malaria, tuberculosis, fever, microbial infections [7], gastrointestinal disorders (constipation and diarrhea), anaemia, and toothache among others [8]. Almost all parts of this tree (fruit pulp, seeds, leaves, flowers, roots, and bark) are used in the traditional African medicine [9,10], whereas, in Europe, only the fruit pulp is consumed as a food since its authorization as a novel food ingredient by the European parliament and council under the Regulation (EC) No. 258/97 (Commission Decision 2008/575/EC). Furthermore, growing literature evidence suggests that *Adansonia digitata* L. can improve carbohydrates and lipids metabolism, exerting hypoglycemic and hypolipidemic activities [11,12,13,14].

In this context, this study aimed to investigate the possible mechanisms of action underlying the beneficial effects of baobab extracts observed in animal model systems. All the different edible parts of the baobab (fruit pulp, leaves, raw seeds, and toasted seeds) are evaluated as possible sources of advantage in reducing the main risk factors of MS. In detail, we tested the effect on the enzymes, which play a crucial role in MS and above-mentioned related pathologies development: alpha-amylase, alpha-glucosidase, angiotensin-converting enzyme (ACE), 3-hydroxy-3-methyl-glutaryl-coenzyme A reductase (HMG-CoAR), and pancreatic lipase.

Thus, the extracts obtained from the edible parts of baobab were assayed by in vitro enzymatic assays to evaluate their inhibitory capacity against these enzymes. As baobab fruit and leaf extracts were found to be the most active, we continued in testing their potential activity on the differentiation process of SW-872 human liposarcoma cells to mature adipocytes. Since these cells can be differentiated to mature adipocytes upon treatment with 100 μM oleic acid for 7 days, they can actually be used as a model for human adipocytes for studying the metabolism of lipids [15,16,17].

## 2. Results

### 2.1. Baobab Edible Part Extracts

As reported in the Materials and Methods section, baobab fruit pulp, leaves and raw and toasted seeds were submitted for extraction. The extracts were freeze-dried and the yield of the extraction procedure was evaluated. Baobab fruit pulp extract gave the highest yield (22.3%), followed by baobab leaves (12.2%), while baobab raw and toasted seeds gave the lowest yield, which was found to be 5.9% and 4.9%, respectively. The chemical characterization was performed in a previous study of Tsetegho et al. (2019) although reverse-phase high-performance liquid chromatography with photodiode array detection coupled to electrospray ion-trap mass spectrometry (RP-HPLC-PDA-ESI-MS/MS analysis) [18].

### 2.2. In Vitro Enzymatic Inhibition Assays

#### 2.2.1. Alpha-Amylase Inhibition Assay

**Figure 1:**
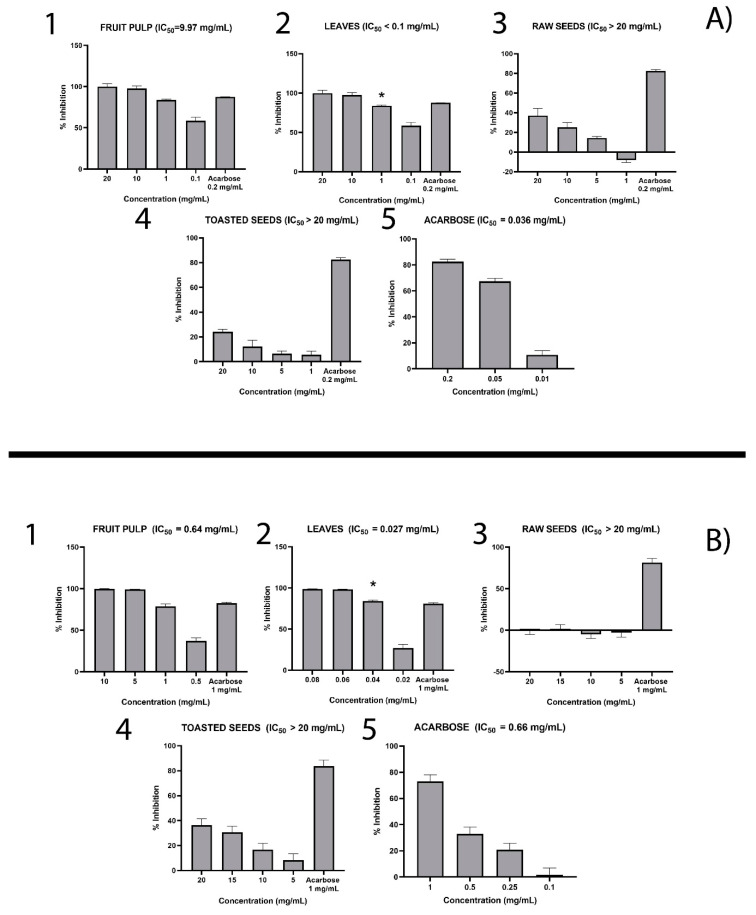
Inhibitory activity of extracts from baobab edible parts towards alpha-amylase (**A**) and alpha-glucosidase (**B**). The results are expressed as mean of percentage (%) inhibition (± SD). Acarbose was used as positive control. 1. Fruit pulp extract; 2. Leaf extract; 3. Raw seed extract 4. Toasted seed extract; 5. Acarbose. IC_50_ values are also reported. * means statistically significant difference (*p* < 0.05) between the two groups.

Figure 1A showed the results obtained from the alpha-amylase inhibition assay.

Acarbose, a known inhibitor of alpha-amylase, inhibited the enzyme in a concentration-dependent manner, with an IC50 of 0.036 mg/mL (Figure 1A5). As far as baobab leaf and fruit pulp extracts are concerned (Figure 1A1,A2), they inhibited alpha-amylase activity in a concentration-dependent manner with IC50 of 0.10 and 9.97 mg/mL, respectively. Baobab leaves showed the most potent activity against alpha-amylase, although this activity was nearly three times lower than that of acarbose. Both raw and toasted baobab seed extracts weakly inhibit alpha-amylase, though the raw seed extract seems to be more active than toasted seeds (Figure 1A3,A4).

#### 2.2.2. Alpha-Glucosidase Inhibition Assay

The results of alfa-glucosidase inhibition assay suggest that acarbose inhibited the enzyme activity in a concentration-dependent manner with an IC50 of 0.66 mg/mL (Figure 1B5). The baobab leaf and fruit pulp extracts also inhibited alpha-amylase in a concentration-dependent manner with IC50 = 0.03 mg/mL and = 0.64 mg/mL, respectively (Figure 1B1,B2). Also, in this case, the baobab leaf extract resulted in being more active than the fruit pulp extracts. In fact, the baobab leaf extract showed the most potent activity against alpha-glucosidase with an IC50 lower than that obtained for acarbose. Both raw and toasted baobab seeds weakly inhibited alpha-glucosidase, though in this case, the toasted seeds appeared to be more active, unlike in the alpha-amylase assay results (Figure 1B3,B4).

#### 2.2.3. ACE Inhibition Assay

**Figure 2:**
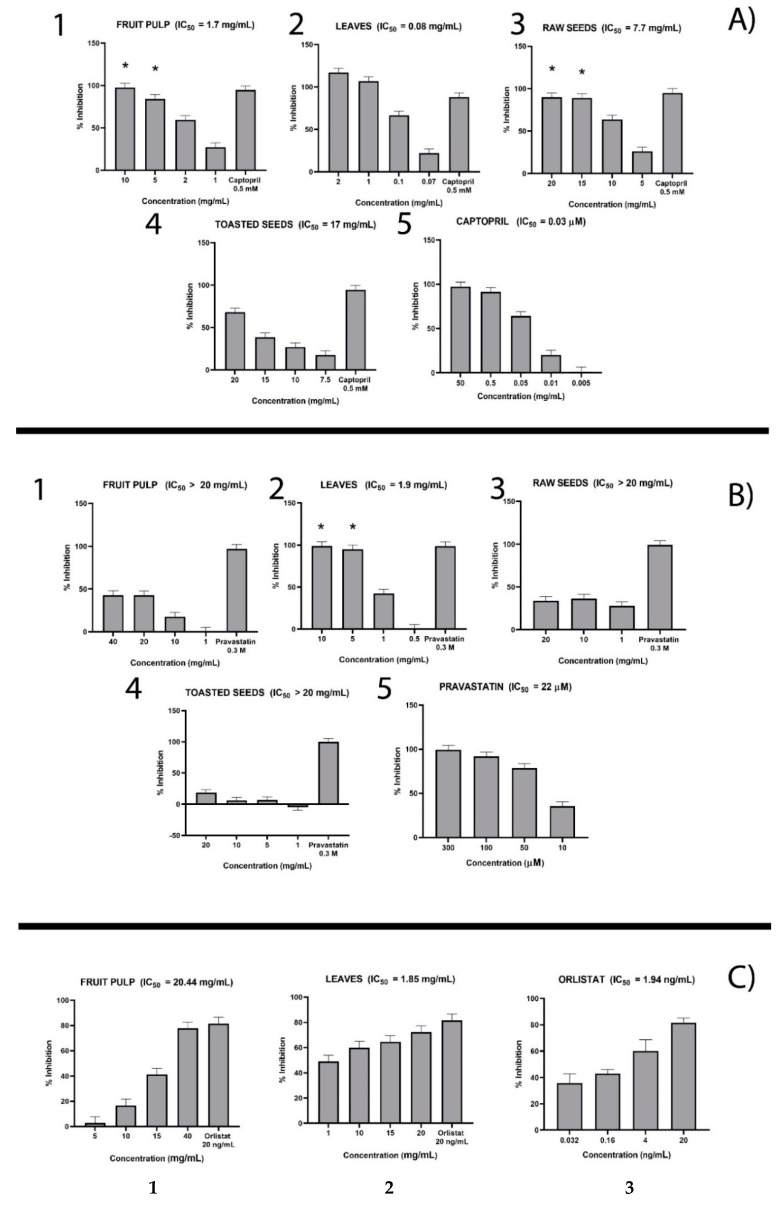
Inhibition activity of extracts from baobab edible parts against ACE (**A**), HMGCoA-R (**B**), and pancreatic lipase (**C**). The results are expressed as mean of percentage (%) inhibition (± SD). Captopril, pravastatin, and orlistat were used as positive control at increasing concentrations. 1. Fruit pulp extract; 2. Leaf extract; 3. Raw seed extract; 4. Toasted seed extract; 5. Pravastatin. IC_50_ values are also reported. * means statistically significant difference (*p* < 0.05) between the two groups.

In ACE inhibition assay, L-captopril was used as positive control. It inhibited the enzyme in a concentration-dependent manner with an IC50 of 30 nM (Figure 2A5).

Under our experimental conditions, all baobab extracts inhibited ACE in a concentration-dependent manner. In detail, the baobab leaf extract (Figure 2A2) showed the highest inhibition (IC50 = 0.08 mg/mL) followed by the fruit pulp (IC50 = 1.7 mg/mL) (Figure 2A1), then the raw seeds (IC50 = 7.77 mg/mL) (Figure 2A3) and the toasted seeds (IC50 = 17 mg/mL) (Figure 2A4).

#### 2.2.4. HMG-CoAR Inhibition Assay

The data obtain to HMG-CoAR inhibition assay is reported in Figure 2B5, where pravastatin used as the positive control inhibited HMG-CoAR in a concentration-dependent manner, with an IC50 of 22.70 μM. In our experimental conditions, the tested baobab extracts (Figure 2B1–4) were found to exert very low inhibitory activity. In detail, baobab leaves showed the highest inhibitory activity against HMG-CoAR with an IC50 of about 1.90 mg/mL, followed by the fruit pulp and raw seed extracts which show an IC50 higher than 20 mg/mL. Baobab raw seed extract showed very low inhibition.

#### 2.2.5. Pancreatic Lipase Inhibition Assay

Orlistat was used as positive control in the pancreatic lipase inhibition. This compound showed dose-dependent inhibition with an IC_50_ = 1.94 ng/mL (Figure 2C3). As far as baobab extracts are concerned, leaf extract (Figure 2C2) showed the most potent inhibitory activity with an IC50 of 1.85 mg/mL followed by baobab fruit pulp (Figure 2C1), which has an IC50 of 20.44 mg/mL. Baobab raw and toasted seed extracts did not show any inhibition of pancreatic lipase (data not shown).

### 2.3. Effects of Baobab Fruit Pulp and Leaf Extracts on the Differentiation of SW-872 Human Liposarcoma Cells to Mature Adipocytes

As baobab fruit and leaf extracts were found to be the most active in terms of enzyme activity inhibition, their effects on the process of adipocyte differentiation of SW-872 cells were tested.

#### 2.3.1. Differentiation of SW-872 Preadipocytes to Adipocytes

**Figure 3:**
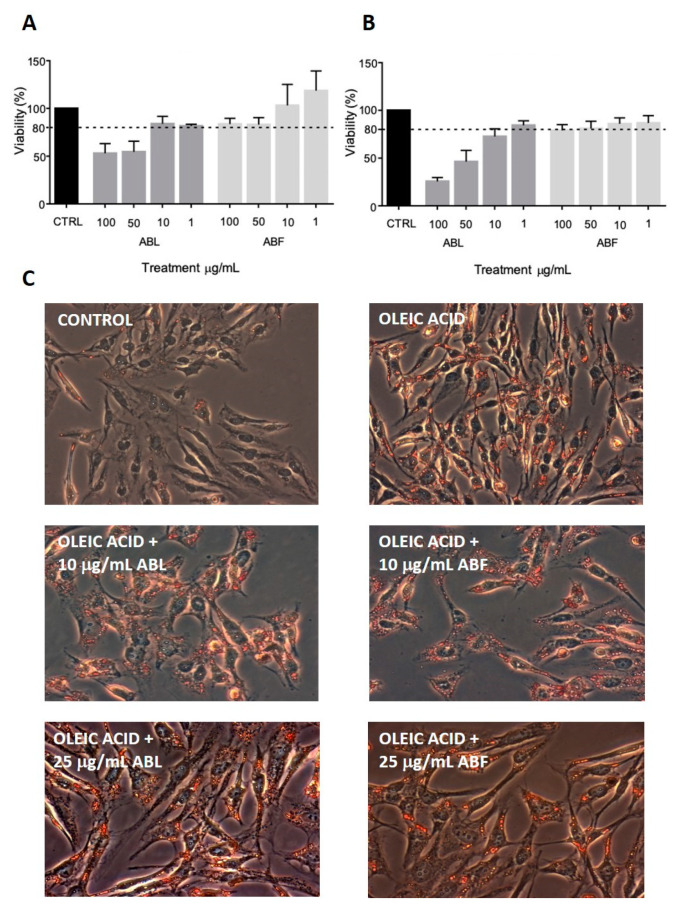
Cytotoxicity activity of extracts from baobab edible parts in SW-872 cells (MTS assay) (**A**,**B**). Cells were differentiated with 100 μM oleic acid for 7 days and then were incubated for 24 (**A**) or 48 h (**B**) with plant extracts. Data are derived from pooling of two separate experiments (total n = 4) and are expressed as mean of percentage (%) inhibition (± SD). Effect of baobab extracts on adipocyte differentiation of SW-872 cells with oleic acid (ORO staining) (**C**). SW-872 cells were incubated for 7 days with 100 µM oleic acid, with or without 10 and 25 mg/mL baobab extracts. The red staining (ORO) indicates relevant triglyceride accumulation (marker of adipocyte differentiation) (inverted phase contrast microscopy; 20X magnification). The addition of 10 or 25 µg/mL baobab extracts to 100 µM oleic acid did not affect triglyceride accumulation. Control: Untreated SW-872 cells (preadipocytes). ABL: Baobab leaf extract; ABF: Baobab fruit pulp extract.

SW-872 preadipocytes were differentiated to adipocytes with 100 μM oleic acid. Figure 3 shows the ORO staining of SW-872 cells under non-differentiated (control) and differentiated (oleic acid) conditions (Figure 3B). The marked red staining in differentiated SW-872 cells indicates the abundant accumulation of TG (triglycerides), as expected in well-differentiated adipocytes.

#### 2.3.2. Effect of Baobab Fruit Pulp and Leaf Extracts on SW-872 Cell Viability

The cytotoxicity of plant extracts was assayed by measuring cell viability with the MTS assay and by morphological analysis. SW-872 cells were treated with increasing concentrations (0–100 μg/mL) of baobab leaf and baobab fruit pulp extracts for 24 and 48 h. The viability threshold was set at 80%. Toxicity was observed at 50 and 100 μg/mL for baobab leaf extract, both at 24 and 48 h (Figure 3A), while at 10 μg/mL, baobab leaf extract only marginally affected cell viability. Baobab fruit pulp extract was not toxic at all concentrations and times considered (Figure 3A). Since baobab leaf extract concentrations below 50 μg/mL resulted as non-toxic, we used this or lower concentrations for further experiments. An additional viability evaluation was conducted by examining cell morphology after treatment with baobab extracts for 24 and 48 h. No morphological changes were observed for both extracts at 1 and 10 μg/mL at both times; whereas, at 100 μg/mL, baobab leaf extract, but not baobab fruit pulp extract, resulted in toxic and severe morphological changes leading to cell death in SW-872 cells after 24- and 48-h treatments (Figure S1 in Supplementary Materials).

#### 2.3.3. Effect of Baobab Fruit Pulp and Leaf Extracts on SW-872 Differentiation

The study of the effects of baobab extracts on the differentiation of SW-872 preadipocytes into mature adipocytes was assessed evaluating TG accumulation by ORO staining and TG content measurement. SW-872 cells were simultaneously treated with 100 µM oleic acid as a differentiating agent, and baobab extracts at 10 and 25 µg/mL, for 7 days. We considered undifferentiated cells as negative control and SW-872 cells treated only with oleic acid as a positive control (Figure 3B). The addition of both baobab extracts at 10 and 25 µg/mL did not affect the extent of ORO staining (Figure 3B). Moreover, the extent of TG accumulation by SW-872 cells in the presence of either baobab extract was also quantified, using the same experimental paradigm reported above for ORO assessment. As expected, TG content in SW-872 cells doubled after 7-day exposure to oleic acid. The simultaneous addition of either baobab fruit extract or baobab leaf extract, however, did not significantly affect TG accumulation (Table 1), indicating a neutral impact of these extracts on this parameter.

### 2.4. Molecular Docking

Recent evidence showed that flavonoids, especially flavonols, exert inhibitory activity against the enzymes studied in this investigation [19,20,21,22]. In Table 2, the characterization of the polyphenol binding sites is reported for alpha-glucosidase, alpha-amylase, 3-hydroxy-3-methylglutaryl-coenzyme A reductase, pancreatic lipase, and angiotensin-converting enzyme.

**Figure 4:**
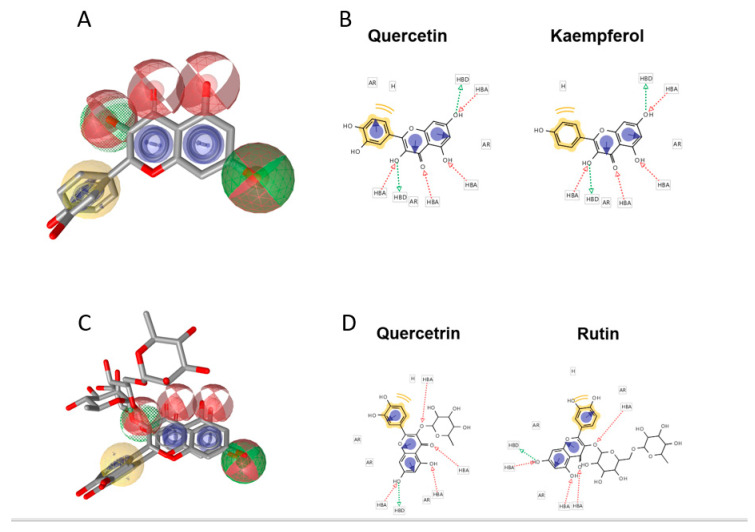
3D and 2D views of the quercetin and kaempferol pharmacophore models respectively (**A**,**B**). 3D and 2D views of the quercetrin and rutin pharmacophore models respectively (**C**,**D**). In the depictions, the features are encoded as AR = aromatic ring in blue.

**Figure 5:**
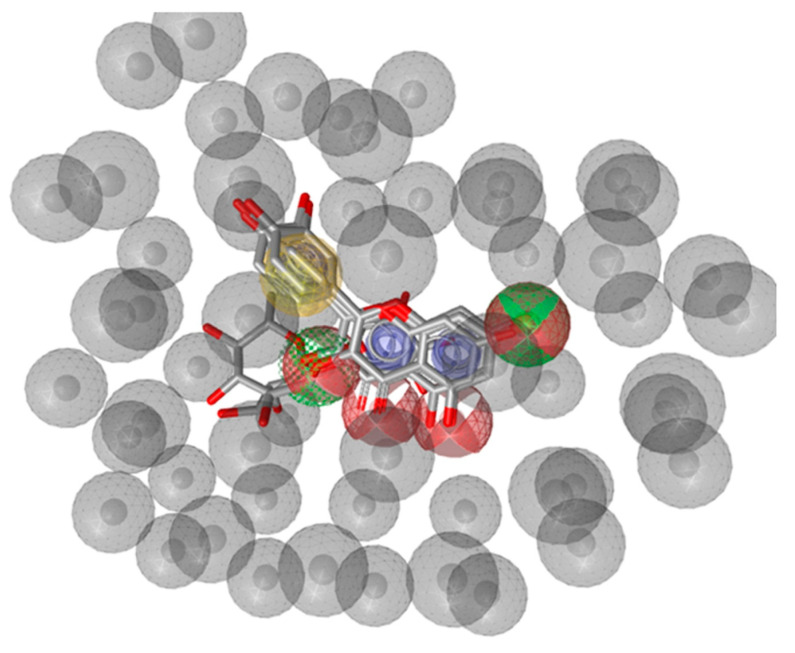
Merged pharmacophore of the compounds quercetin, kaempferol, quercitrin and rutin with features HBA (red spheres), HBD (green spheres) hydrophobic region (yellow sphere), aromatic ring (blue sphere), and volume excluded (gray spheres).

In light of this, and considering that quercetin, kaempferol, and their derivatives occur both in baobab fruit pulp extract and in leaf extract, a pharmacophore model using flavonols available in the baobab extracts was built to evaluate the pattern of common chemical features essential to biological activity against potential targets. The pharmacophore generated by Ligand Scout was built by a training set of four flavonols: quercetin, kaempferol, quercetrin, and rutin. It showed four main features: (1) hydrogen bond acceptors (2) hydrogen bond donors, (3) aromatic rings, and (4) hydrophobic regions. All the ligands highlighted consistency in these four features. In Figure 4, 3D, and 2D views of the quercetin/kaempferol and quercitrin/rutin aligned pharmacophores were depicted. The features identified in red and green colors are the H-bond donor and acceptor groups respectively; the aromatic rings are shown in blue color and hydrophobic in yellow color in both views. Overall, the pharmacophoric features for each are shown in Table S1 (Supplementary Material). Similar features were identified after analyzing the pharmacophores of all compounds generated by Ligand scout. The similar features of all compounds were then superimposed and merged into a single pharmacophore as shown in Figure 5. Excluded volume spheres to reflect potential steric restrictions were also added.

## 3. Discussion

Since MS is a complex cluster of metabolic abnormalities, we decided to test extracts of baobab edible parts to verify their ability to inhibit five enzyme activities involved in the development of MS itself. Baobab extract inhibitory activities obtained by in vitro enzymatic assays were compared with those of anti-hyperglycemic, antihypertensive, anti-hypercholesterolaemic, and anti-obesity drugs, used as positive controls to check the validity of these enzymatic tests. According to the findings obtained, the baobab fruit pulp and leaf extracts appeared to be the most interesting ones. On the contrary, the seed extracts, both raw and toasted, did not show any relevant activity against these enzymes, except for baobab raw seed extract, which showed good inhibitory activity against ACE. This result suggests proceeding with further investigations, as plant extracts with antihypertensive activity are limited in number and baobab seeds could be a source of extracts with such activity.

As far as baobab leaf extract is concerned, it was found to be the most active in all the enzymatic assays. Our results are in agreement with literature data showing that baobab leaves possess potent inhibitory activity against alpha-amylase and alpha-glucosidase enzymes and provide new knowledge about the potential use of baobab leaf extract as an antihypertensive and antidyslipidemic agent [23]. Furthermore, these results provide an explanation of the mechanism of action underlying the evidence obtained by in vivo studies on *Adansonia digitata* L. against MS, with baobab fruit pulp improving glycemic response and satiety [24], reducing serum lipids [25], and maintaining glycemic control in animals [11,26]; moreover, baobab leaf extract is able to reduce hyperglycemia and hyperlipidemia of diabetic rats [14].

Baobab leaf and fruit pulp extracts, at non-toxic concentrations, did not affect TG accumulation in SW-872 cells, taken here as an index of adipocyte differentiation, and thus, they did not reduce or contrast adipogenic differentiation of SW-872 preadipocytes. It should be considered that in this study adipogenesis was induced by oleic acid treatment, which is actually a potent adipogenic promoter that acts via activation of nuclear PPARγ (peroxisome proliferator-activated receptor gamma), and thus, these plant extracts may not be sufficiently effective in counteracting such molecular processes. In any case, this observation also suggests that these extracts are not adipogenic in vitro. This requires further studies in experimental animals and in humans, where they are not expected to promote obesity nor to worsen pre-existing obesity.

In our previous investigation on baobab fruit pulp and baobab leaf extracts [18], we found many flavonols (quercetin, kaempferol, and their derivatives) detected by targeted RP-HPLC-PDA-ESI-MS/MS analysis. The results obtained from the enzyme inhibition assays can be explained considering the binding ability of the flavonols occurring in the baobab fruit pulp and leaf extracts towards the enzymes. This hypothesis is supported by Literature data. Jhong et al. in a research communication using a structure-based docking approach [19], screened 47 natural compounds against alpha-glucosidase and alpha-amylase 3D crystal structures. The docking data showed that flavanols such as catechin, quercetin, and rutin had binding ability towards alpha-amylase and alpha-glucosidase enzymes. Analyzed alpha-glucosidase and alpha-amylase inhibitory activity revealed that the most interesting inhibitors (below 0.5 mM) were flavonoids, and among these, quercetin and catechin were the most potent, in comparison with the commercial drug acarbose. These compounds were found to favorably bind the alpha-glucosidase active site, forming key interactions with R407, R197, R407, and F282 binding residues, similarly to the positive control acarbose. The top-ranked compounds scoring highly on the alpha-amylase crystal structure, including catechin, quercetin, rutin, and other polyphenols, were found to fit the alpha-amylase binding site by a network of H-bonds and electrostatic interactions involving K200, E233, D300, W151, A198, and L162 binding site residues.

To explain polyphenol anticholesterol activities, flavonoids such as kaempferol, catechin, and quercetin can bind to HMG-CoAR [20]. The X-ray crystal structure of human HMG-CoAR reveals a HMG-CoA binding loop comprising of amino acid residues 682–694, the catalytic site of the enzyme. Crucial interaction residues involved K691, E559, and D767. However, the NADP allosteric cleft is also present in HMG-CoAR located near to the active site. Docking studies reported that the rigid ring of phenols can fit the NADP binding site, competitively inhibiting NADPH and preventing HMG-CoA access by steric effects. Recently, Martinez Gonzales et al. predicted the potential binding sites of polyphenols in the pancreatic lipase binding cleft by conducting docking studies [21]. Moreover, some studies have demonstrated that certain flavonoids can have inhibitory effects on different zinc metalloproteinases such as ACE activity, which exhibited a key role in the regulation of arterial blood pressure [27,28]. In 2012, Guerrero et al. [22] used SAR studies to describe the combinations of substructures and functional groups on the flavonoid skeleton, which increase ACE activity. Moreover, docking studies elucidate the molecular mechanism of these compounds on ACE activity. The results highlighted how these flavonoids form intermolecular interactions with some of the ACE binding site regions (i.e., S2′, S2′/S1′ and S1′), that are also involved in the binding of known ACE inhibitors. Interestingly, flavonoids fit the S1 site of ACE, forming most of the intermolecular interactions as found for synthetic ACE inhibitors (e.g., lisinopril, enalapril, captopril). The authors described for example quercetin, rutin and kaempeferol contacted S2′ pocket T282, H353 binding residues, and S2′/S1′ region V379.

## 4. Materials and Methods

### 4.1. Materials and Reagents

Millipore-grade water was obtained from a LC-Pak™ Millex system (Millipore Corporation, Billerica, MA, USA). Formic acid, methanol, alpha-amylase, sodium chloride, sodium phosphate dibasic, sodium phosphate monobasic, starch, dinitrosalicylic acid, acarbose, alpha-glucosidase, 4-nitrophenyl-α-d-glucopyranoside, trichloroacetic acid, tris HCl, HMG-CoAR inhibition assay kit, ACE, *N*-[3-(2-Furyl)acryloyl]-l-phenylalanyl-glycyl-glycine, captopril, porcine pancreatic lipase, p-NPB, dimethylformamide, orlistat, and (3-(*N*-morpholino) propanesulfonic acid were purchased from Sigma-Aldrich, St. Louis, MO, USA.

### 4.2. Preparation of Food Materials and Food Extracts

To perform a more representative sampling, five fresh baobab fruits (about 300 g), and 20 dried leaves, raw and toasted (in an electric oven for 10 min at 240 °C) seeds (500 g) were collected during the harvest season of January–February 2018, in a local market in Ngaoundéré, Cameroon. Baobab fruit pulp samples were oven dried at 40 °C, milled, and mixed together. Baobab dried leaves were collected and milled together. Baobab dried fruit pulp, dried leaves, and raw and toasted seeds were transported to Italy by airplane in plastic bags inside a box, where they were stored in vacuum plastic bags away from sunlight in a cool room. Raw and toasted seeds were crushed in a mortar, ground, and mixed. All the edible parts of baobab were submitted to extraction, which was carried out separately by weighing 10 g of the food powder into a conical flask and then adding 100 mL of a mixture of acidified Millipore grade water with formic acid (0.1%) to methanol (50:50 *v/v*). The conical flask, immersed in an ice bath, was left stirring for 24 h under a nitrogen atmosphere. After 24 h, the mixture was filtered under vacuum with paper filters (Whatman Grade 595 Qualitative Filter Papers), and the methanol was evaporated under a stream of nitrogen. Finally, the obtained extract was frozen at −20 °C and then freeze-dried to obtain a powder. The yields of extraction are reported above. The different extracts were dissolved in the different buffers for the enzymatic assays. In our recent study of Tsetegho et al. (2019), the baobab food extracts were characterized though RP-HPLC-PDA-ESI-MS/MS and NMR spectroscopy to obtain a phytochemical profile [18].

### 4.3. In Vitro Enzymatic Assays

The enzyme inhibition assays were conducted according to the protocol described in the Worthington enzyme manual, with some modifications [29]. For the alpha-amylase inhibition assay, each food extract was diluted in a range of 0.01 to 40.00 mg/mL. For alpha-glucosidase inhibition assay, each food extract was diluted in a range from 0.02 to 20.00 mg/mL. Acarbose was used as a positive control in the concentration range of 0.20–0.01 mg/mL in both assays. For the HMG-CoAR inhibition assay, the extracts were tested in a range from 0.50 to 40.00 mg/mL. Pravastatin was used as a positive control. For the pancreatic lipase inhibition assay, each extract was diluted in a range from 1.00 to 40.00 mg/mL. Orlistat was used as a positive control. For ACE inhibition assay, each extract was diluted in 50 mM Tris-HCl buffer (pH 7.5 with 0.3M NaCl) in a range from 0.07 to 20.00 mg/mL. Captopril was used as a positive control. For all enzyme inhibition assays, the absorbance of sample blanks (buffer in place of the enzyme solution) and a control (buffer in place of plant extract solution) were recorded as well. The final sample absorbance was obtained by subtracting the corresponding absorbance of the sample blank. The inhibition of enzyme activity was calculated according to the equation below:

Inhibition (%) = ((Abs Control-Abs sample)/(Abs Control)) × 100(1)

For all assays, experiments were performed three times (3 replicates).

The IC50 values were calculated using linear or polynomial regressions. Logarithmic transformations were used to facilitate the calculations where necessary. Results are expressed as means ± SE, and *p* < 0.05 was considered statistically significant. The statistical significance of the data was assessed through a one-way variance analysis (ANOVA). When significant differences were found, Bonferroni post hoc testing was used to determine the difference between the groups involved.

### 4.4. Evaluation of the Effects of Baobab Pulp and Leaf Plant Extracts on SW872 Human Liposarcoma Cells Differentiated to Mature Adipocytes

#### 4.4.1. SW-872 Cell Culture

The SW-872 human liposarcoma cell line (ATCC^®^ HTB-92 ™) was obtained from the American Type Culture Collection (ATCC^®^, Manassas, VA, USA) and grown as recommended by the latter. Cells (undifferentiated preadipocytes and differentiated adipocytes) were cultured in DMEM/F-12 culture medium (Dulbecco’s Modified Eagle Medium: Nutrient Mixture F-12), containing 15 mM of HEPES buffer (*N*-[2-hydroxyethyl]piperazine-*N*′ [2-ethanolsulfonic acid] and sodium bicarbonate, to which 2 mM l-glutamine, 10% fetal bovine serum (FBS) and 1% penicillin (100 U/mL), and streptomycin (100 μg/mL) were added. They were then kept in culture in 75 cm^2^ Petri dishes, at 37 °C at 95% air and 5% CO_2_. The culture medium was renewed every 2 days. After 7 days of culture, 2 mL of a trypsin-EDTA solution was added to the plate to detach the cells and then incubated at 37 °C for 5 min. Subsequently, the trypsin-EDTA-cell suspension was centrifuged at 1,200 rpm for 5 min to separate the cells. The cell pellet was resuspended in 10 mL of medium containing 10% FBS.

#### 4.4.2. Differentiation of SW-872 Preadipocytes to Mature Adipocytes

SW-872 preadipocyte cells were cultured as described above. After 24–48 h, oleic acid was added directly to the plate to a final concentration of 100 μM. Medium change and addition of oleic acid were repeated every 48 h. The differentiation treatment was prolonged for 7 days. After this treatment, adipocytes maintained their differentiated state for at least 4 days without adding further oleic acid to the medium.

#### 4.4.3. Oil red O (ORO) Staining/Hematoxylin Counterstaining of Nuclei

After removing the medium, cells were washed with PBS and fixed with 4% formaldehyde for 60 min and washed three times with water. Then, cells were incubated with the ORO working solution (3 mg/mL) (Sigma-Aldrich, Milan, Italy) for 30 min. Cells were then washed 10 times with water, nuclei were counterstained with hematoxylin, and the morphology was evaluated by immunofluorescence microscopy.

#### 4.4.4. Triglyceride Content Measurement

One of the main features determining the differentiation from preadipocytes to mature adipocytes is the accumulation of triglycerides in the cell. To this aim, we used the Triglyceride Quantification Kit (Sigma-Aldrich, Milan, Italy), which provides a sensitive assay to measure triglyceride (TG) concentration, according to the protocol provided by the supplier. Briefly, in this assay, TGs are converted to free fatty acids and glycerol. Glycerol was then oxidized to generate a colorimetric (570 nm)/fluorometric (λex = 535 nm/λem = 587 nm) product. The kit is sensitive to detect 2 pmole–10 nmole (2–10,000 μM range) of TG. The protocol was adapted for use with SW-872 cell extracts and all samples and standards were prepared in duplicate. Data were corrected for the background and interpolated in an appropriate TG standard curve.

### 4.5. Ligand-Based Pharmacophore Modeling

The pharmacophore models were built using LigandScout 4.3 (InteLigand, Wien, Austria) from a training set of four flavonol structures retrieved from the PubChem Repository [29]. For the training set, 25 conformations were created with OMEGA-best settings implemented in LigandScout [30]. Successively, the conformations were clustered according to the similarity of the pharmacophore radial distribution function (RDF). The program was set to create 10 shared feature pharmacophore hypotheses ranked according to pharmacophore fit and atom overlap scoring function. In a shared feature pharmacophore model generation, LigandScout generates pharmacophore models from the chemical functionalities of the training compounds and aligns the molecules according to their pharmacophores [31].

Only features present in all training molecules are considered for model building. For the best alignment, common pharmacophore features are generated and assembled together, comprising the final pharmacophore model. The shared feature pharmacophore models contain only chemical features present in all the training molecules.

## 5. Conclusions

Currently, many drugs are used for their inhibitory activities against the enzymes involved in the pathogenesis of MS. Most of these drugs present many side effects and are expensive. Thus, alternative options are required to face these issues. Food extracts including plant extracts are very complex and contain several active components, offering a higher probability of finding various components that may target different metabolic pathways, thus suggesting the utility of food extracts in complex pathogenic processes, like MS, which involves many enzymes and metabolic pathways. The novel data discussed in the present paper may thus help to support and explain the growing amount of evidence on the nutritional and biological properties of baobab edible parts and provide suggestions on their possible uses in food and nutraceutical fields, prompting European food companies to present an application for authorization of a novel food in the context of Regulation (EU) 2015/2283, as already done in the case of the baobab fruit (Regulation (EC) No. 258/97 (Commission Decision 2008/575/EC).

## Figures and Tables

**Table 1:** Effect of baobab leaf extract and baobab fruit pulp extract on triglyceride accumulation in SW-872 cells differentiated to adipocytes with 100 µM oleic acid (n = 3).

Cell Treatment	mg/mL (Mean (SD))	%
Control	1.318 (0.131)	100
Oleic acid	2.821 (0.660)	214
Oleic acid + baobab leaf extract	3.158 (0.281)	240
Oleic acid + baobab fruit pulp extract	3.005 (0.213)	228

**Table 2:** Polyphenol binding sites characterization.

Molecular Target	Catalytic Site	Allosteric Site	Residues Involved	Ref.
Alpha-glucosidase	√		R407, R197, R407, F282	Jhong et al., 2015 [19]
Alpha-Amylase	√		K200, E233, D300, W151, A198, L162	Jhong et al., 2015 [19]
3-Hydroxy-3-methylglutaryl-coenzyme A reductase (HMGCoAR)		√ (NADP binding site)	K691, E559, D767	Islam et al., 2015 [20]
Pancreatic Lipase	√		F78, Y115, H152, F216	Martinez Gonzales et al., 2017 [21]
Angiotensin-converting enzyme (ACE)	√		S1′, S2′/S1′, S2′ regions	Guerrero et al., 2012 [22]

## Supplementary Materials

The following are available online. Table S1: Pharmacophore features of the flavanols: quercetin, kaempferol, quercitrin, rutin. Figure S1. Morphological study of SW-872 cells, differentiated with 100 μM oleic acid, after 48 h treatment with plant extracts.
