# Supplementary material for: Hydromethanolic Extracts from Adansonia digitata L. Edible Parts Positively Modulate Pathophysiological Mechanisms Related to the Metabolic Syndrome
Source: Molecules. 2020 Jun 21;25(12):2858. doi: 10.3390/molecules25122858 (PMC7356617; doi:10.3390/molecules25122858)
Supplement: Supplementary file 1 [file molecules-25-02858-s001.pdf]

# Supplementary Material

**Table S1**. Pharmacophore features of the flavanols:quercetin, kaempferol, quercitrin, rutin

| Compound name | HBA | HBD | Hydrophobic region | Aromatic ring |
|---|---|---|---|---|
| Quercetin | **4** | **2** | **1** | **3** |
| Kaempferol | **4** | **2** | **1** | **2** |
| Quercetrin | **4** | **1** | **1** | **3** |
| Rutin | **4** | **1** | **1** | **3** |

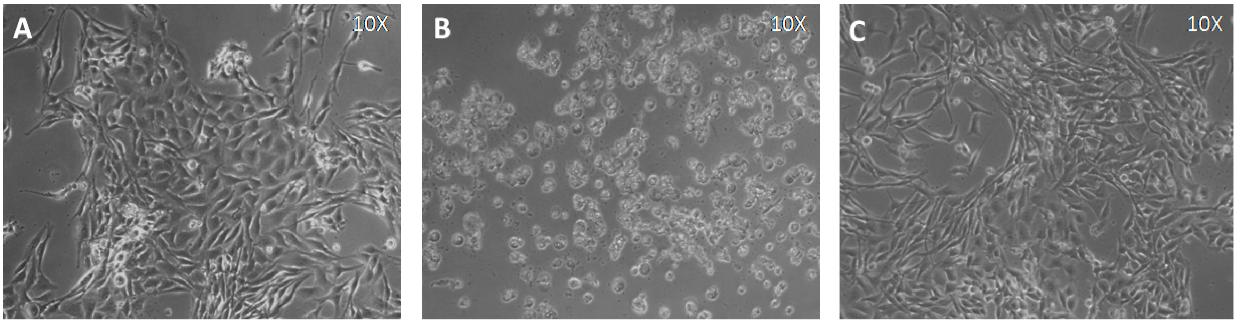


**Figure S1.** Morphological study of SW-872 cells, differentiated with 100 M oleic acid, after 48 h treatment with plant extracts. A. Control cells were well adherent, displaying a normal morphology. B. Cells treated with 100 µg/mL ABL extract. C. Cells treated with 100 µg/mL ABF extract. In panel B, cells show severe morphological changes leading to cell death, including rounding and shrinking of the cells, disintegration of the membranes and cytoplasmic aggregation; in panel C, cells mostly show a normal morphology, although in some cells, initial features of cell damage can be appreciated (inverted phase contrast microscopy; 10X magnification).
